# The role of corporate-academic research partnerships in bringing novel therapeutics and diagnostics to the market

**DOI:** 10.1186/1479-5876-10-S2-A42

**Published:** 2012-10-17

**Authors:** William Rhodes

**Affiliations:** 1 Corporate Strategy and Development, BD, USA

## 

Healthcare industry participants – whether biotech, diagnostic, medical device or classical pharmaceuticals – depend in large part on successfully collaborating with academic institutions as a source of innovation and clinically-relevant new products. Historically this has most often been in the form of pure licensing arrangements, whereby new inventions and discoveries are licensed by the manufacturer for their internal further development and eventual marketing, and royalty payments accrue to the institution. In this model, the ‘discovery’ is the providence of the academic researcher, and the risk and cost of development is borne by the manufacturer.

Increasingly, however, collaborative partnerships are forming between industry and academia that encompass a broader scope, longer duration, and involve greater risk sharing. A major driver of these collaborative activities is the increasing recognition that translational medicine – linking discovery with clinical application – is a means for both the industrial and academic partner to reduce their respective risks, quickly demonstrate new product safety and efficacy and more rapidly bring new treatment and diagnostic modalities to market.

Prime examples of this new paradigm are now being created in the areas of cell therapy and personalized medicine, particularly companion diagnostics. In this talk, we will discuss these examples, the new model that is developing, and how both academic institutions and industry can form successful partnerships.

